# Atypical lymphocyte count correlates with the severity of dengue infection

**DOI:** 10.1371/journal.pone.0215061

**Published:** 2019-05-01

**Authors:** Choong Shi Hui Clarice, Visula Abeysuriya, Sanjay de Mel, Basuru Uvindu Thilakawardana, Primesh de Mel, Chandima de Mel, Lal Chandrasena, Suranjith L. Seneviratne, Christina Yip, Eng Soo Yap

**Affiliations:** 1 Department of Haematology-Oncology, National University Cancer Institute, National University Health System Singapore, Singapore, Singapore; 2 Nawaloka Hospital Research and Education Foundation, Nawaloka Hospitals PLC, Dharmadasa Mawatha, Colombo, Sri Lanka; 3 Department of Surgery, University of Colombo, Colombo, Sri Lanka; 4 Institute of Immunity and Transplantation, Royal Free Hospital and University College London, London, United Kingdom; 5 Department of Laboratory Medicine, National University Health System Singapore, Singapore, Singapore; Royal College of Surgeons in Ireland, IRELAND

## Abstract

**Introduction:**

The early identification of patients at risk of severe dengue infection (DI) is critical to guide clinical management. There is currently no validated laboratory test which can predict severe complications of DI. The Atypical lymphocyte count (ALC) is a research parameter generated at no extra cost when an automated Full Blood Count (FBC) is performed. The purpose of this study was to assess the association of ALC with the severity of DI.

**Methods:**

We prospectively collected data on patients admitted to Nawaloka Hospital Sri Lanka (NH) with DI between December 2016 and November 2017. DI was diagnosed based on a positive Non-structural antigen 1 (NS1) or dengue IgM antibody. ALC (absolute ALC and percentage) data were extracted from the Sysmex XS500i automated full blood count (FBC) analyzer (Sysmex Corporation Kobe, Japan). Clinical data was recorded from medical records and the computerized data base maintained by NH.

**Results:**

530 patients were enrolled. Patients with clinical manifestations of severe dengue have a significantly higher AL % compared to dengue without warning signs. Patients who presented with respiratory compromise had statistically significantly higher AL% compared to those without. (AL%; 8.65±12.09 vs 2.17±4.25 [p = 0.01]). Similarly, patients who developed hypotension had higher AL% compared to those who did not suffered from shock (AL%; 8.40±1.26 vs 2.18±4.25 [p = 0.001]). The AL% of dengue patients presenting with bleeding, at 4.07%, is also higher than those without bleeding complications, at 2.15%. There was a significant negative association between platelet count and AL% (p = 0.04).

**Conclusions:**

Clinical manifestations of severe dengue have a significantly higher AL % compared to dengue without warning signs. AL % at presentation may be predictive of severe DI and future larger prospective longitudinal studies should be done to determine if AL % on admission is predictive of the complications of DI.

## Introduction

Dengue infection (DI) is the most common arthropod-borne virus disease, with 2.5 billion people worldwide, across all age groups, at risk of infection [1]. The clinical manifestations of DI are heterogeneous, with the most severe and life-threatening forms being dengue hemorrhagic fever (DHF) and dengue shock syndrome (DSS)[2]. Between 2001 to 2010, an estimated 5,906 DI related deaths were reported[3].

Severe complications of DI occur in only approximately 2–5% of patients, but it is important to be able to identify patients who will develop complications early to assist in effective risk stratification. [4,5]. Early risk stratification of patients with DI can potentially prevent overburden of the healthcare system by focusing on those who need expert care and those who do not, especially in resource limited countries[6]. However, there is currently no validated laboratory test which can predict severe complications of DI. Several clinical parameters have been proposed as predictive factors of severe DI. These include the presence of abdominal pain, vomiting, nausea, hepatomegaly, fluid accumulation, bleeding, respiratory distress, altered mental status and shock[7]. However, these clinical parameters can be subjective as they are based on clinical judgement [8].

The diagnosis of DI is based on clinical history and laboratory tests. The most widely used diagnostic test for DI is an enzyme linked immunosorbent assay (ELISA) [9] which measures dengue Ig M or Ig G antibodies. This test can only be reliably detecting dengue antibodies 3–4 days after the onset of symptoms; hence, false negative results remain a concern. The alternatives are the use of the dengue nonstructural protein 1 (NS1) antigen, which is a glycoprotein necessary for the viability of dengue virus; and the dengue reverse transcriptase PCR (RT-PCR). Although these tests are useful for the diagnosis of DI, they are not useful for the prediction of DI severity [10].

The Atypical Lymphocyte count (AL) is a research parameter generated when a Full Blood Count (FBC) is performed on Sysmex automated FBC analyzers. This technology is known to be specific and sensitive for the detection of abnormal white blood cells such as atypical lymphocytes[11]. These atypical lymphocytes have an increased nucleic acid percentage and characteristics scatter properties which can be detected by an increase in fluorescence signal[12]. During DI progression, it has been observed that affected patients produced atypical lymphocytes seen in the peripheral blood circulation. These atypical lymphocytes have been identified as CD19+ B lymphocytes using flow cytometry[13]. It is postulated that these lymphocytes are antibody immune reaction to the dengue virus, which could explain the significant increase in anti-dengue immunoglobulin G (IgG) antibodies during the secondary dengue infection. Recently, atypical plasmacytoid lymphocytes have been described in patients with dengue infection, which similarly correlates to the significant elevation in IgG levels[14]. AL has been demonstrated to be useful for the differentiation of DI from other viral infections [11, 12]. Therefore, we seek to assess the association of AL with severity of dengue infection.

## Methodology

### Study design

We conducted a prospective observational cohort study on adult patients admitted to Nawaloka Hospital Sri Lanka (NH) with DI between 2016 December and 2017 November.

### Ethical approval

Ethical approval for this study was obtained from the Ethics Review Committee, Faculty of Medicine, and University of Colombo. All patients provided informed consent for their data to be included in the study.

### Study population

530 patients aged 18 to 80 years with DI confirmed by a positive NS1 antigen and/or positive dengue immunoglobulin M (Dengue IgM) ELISA (enzyme linked immunosorbent assay; CE-CTK biotech test kits) were included in this study. Patients with a known history of hematologic malignancy, immunosuppression, HIV positive status, known infection with other viral pathogens were excluded.

### Data collection

AL data were extracted from the Sysmex XS500i automated FBC analyzer (Sysmex Kobe Japan) on the first full blood count (FBC) drawn as part of the patients’ routine investigations. Clinical parameters including presenting symptoms, vital signs, results of other blood investigations and clinical progress (specifically the development of severe DI) were recorded by a study team member during the patients’ admission.

We categories dengue fever according to the 2009 WHO criteria classify dengue according to levels of severity: (A) dengue without warning signs; (B) dengue with warning signs (abdominal pain, persistent vomiting, fluid accumulation, mucosal bleeding, lethargy, liver enlargement, increasing hematocrit with decreasing platelets); and (C) severe dengue (dengue with severe plasma leakage, severe bleeding, or organ failure) [7].

### Data analysis

Data were entered into EXCEL worksheets and checked manually and corrected where necessary. Descriptive statistics were derived and expressed as measures of central tendency and frequencies. Hence, Atypical Lymphocyte % distribute normally P-values obtain through student t-test was used to compare averages between two groups. ANOVA was used to compare mean values of more than two groups. The predictive cutoff value was obtained through Receiver Operating Characteristics (ROC) curve analysis. Data were analyzed using the Statistical Package for Social Sciences 16 (SPSS) (SPSS 16.0, Chicago, Illinois, USA) and STATA version 12 (12.0, Texas, USA). P values of <0.05 were considered significant.

## Results

Table 1 shows the socio-demographic characteristics of study population. 52.5% of patients were female. The median age was 34 years. 77.9% did not have any co-morbidity. 43.7% of the patients were admitted to hospital following two days of fever, while 84.3% were admitted after four days of fever. Confirmation of DI was based on positive Dengue NS1 (81.1%), Dengue IgM (16.9%) and both (2.0%). The percentage of patients who suffered from severe dengue is 1.32%.

**Table 1:** Socio demographic characteristics of study population.

Variable	Number	Percentage %
**Age (In years)**		
Less than 20	42	7.9
21 to 40	278	52.4
41 to 60	175	33.0
Above 61	35	6.7
**Sex**		
Female	278	52.5
Male	252	47.5
**Co-morbidity**		
None	413	77.9
Diabetes	23	4.3
Hypertension	30	5.6
Hyperlipidemia	36	6.9
Other	27	5.3
**Days of fever on admission**		
Less than 2	232	43.7
3 to 4	217	40.6
5 to 6	55	10.4
More than 7	26	5.3
**Confirmation of Dengue**		
Dengue NS1 Positive	430	81.1
Dengue IgM Positive	90	16.9
Both Positive	10	2.0

The mean total white cell count of the study population was 4.12±2.1 ((х10^9^). Mean (SD) Atypical Lymphocyte Count (ALC) (х10^3^), Atypical Lymphocyte percentage (AL %) and Platelet count (х10^9^) of the cohort were 0.1±0.3, 2.35±4.7 and 83.1±56.9 respectively.

Table 2 shows the parameters of severity of DI among the study population. Patients who presented with respiratory compromise (oxygen saturation less than 95%) had statistically significantly higher AL% compared to those without. (AL%; 8.65±12.09 vs 2.17±4.25 [p = 0.01]). Similarly, patients who developed hypotension (mean arterial pressure less than 65mmHg) had higher AL% compared to those who did not suffered from shock (AL%; 8.40±1.26 vs 2.18±4.25 [p = 0.001]). The AL% of dengue patients presenting with bleeding, at 4.07%, is also higher than those without bleeding complications, at 2.15%. There were no deaths reported among the study population.

**Table 2:** Parameters of severity of dengue fever among the study population.

Variable	Number (%)	Atypical Lymphocyte %	P Value*
**Presence of abdominal pain and tender**			
No	411 (77.6)	1.97±3.89	0.18*
Yes	119 (22.4)	2.87±5.24	
**Presence of hepatomegaly of more than 2cm**			
No	516 (97.4)	2.23±4.31	0.16*
Yes	14 (2.6)	2.24±2.31	
**Persistent vomiting despite symptomatic treatment**			
No	515 (97.2)	2.21±4.23	0.81*
Yes	15 (2.8)	1.27±1.30	
**Aspartate transaminase (AST) and Alanine transaminase (ALT) more than 1000 IU/L**			
No	526 (99.3)	2.06±4.68	0.78*
Yes	4 (0.7)	2.50±4.77	
**Presence of respiratory compromise (Oxygen saturation less than 95%)**			
No	517 (97.6)	2.17±4.25	0.01*
Yes	13 (2.4)	8.65±12.09	
**Presence of Shock (Mean arterial pressure less than 65mmHg)**			
No	526 (99.3)	2.18±4.25	0.001*
Yes	4 (0.7)	8.40±1.26	
**Presence of Bleeding**			
No	523 (98.7)	2.15±4.22	0.02*
Yes	7 (1.3)	4.07±5.79	

*Based on Mann-Whitney U Test

Table 3 shows the association between AL % with dengue without warning signs, with warning signs and severe dengue. Based on ANOVA analysis there was statically significant association between three groups (p = 0.001). The highest mean AL% was reported in the severe dengue group (AL%:10.8±3.31).

**Table 3:** Association between atypical lymphocyte percent with dengue without warning signs, with warning signs and sever dengue.

Classification	Number (%)	Atypical lymphocyte %	Significant*
Dengue without warning signs	364 (68.6)	1.59^a^±1.36	P = 0.001 F = 41.84 df = 2
Dengue with warning signs	152(28.6)	4.47^b^±1.56	
Severe dengue	14(2.8)	10.80^c^±3.31	

*Based on ANOVA and

^a, b and c^ Post hoc test (Tukey HSD)

Association between AL % with severe dengue and non-severe dengue (with/without warning sings) shows in Table 4. Based on t-test there was a significant association between two groups. (p = 0.001).

**Table 4:** Association between atypical lymphocyte % with severe dengue and non-severe dengue (with/without warning signs).

Classification	Number (%)	Atypical lymphocyte %	Significant*
Severe dengue	14(2.8)	10.80±3.31	P = 0.001
Non-severe dengue	516(97.2)	2.17± 1.91	

*Based on student t-test

Table 5 shows the association between AL% and mean platelet count (10^9^). Based on ANOVA analysis there was a significant association between AL % categories and mean platelet count (p = 0.04).

**Table 5:** Association between AL% and mean platelet count (10^9^).

AL % Category	Number (%)	Platelet count (10^9^) Mean±SD	Significant*
Below 10	451(85.1)	83.6±47.6	P = 0.04 F = 4.121 df = 2
11 to 20	55(10.3)	52.3±31.6	
Above 21	24(4.6)	47.9±18.1	

*Based on ANOVA test

**Fig 1:**
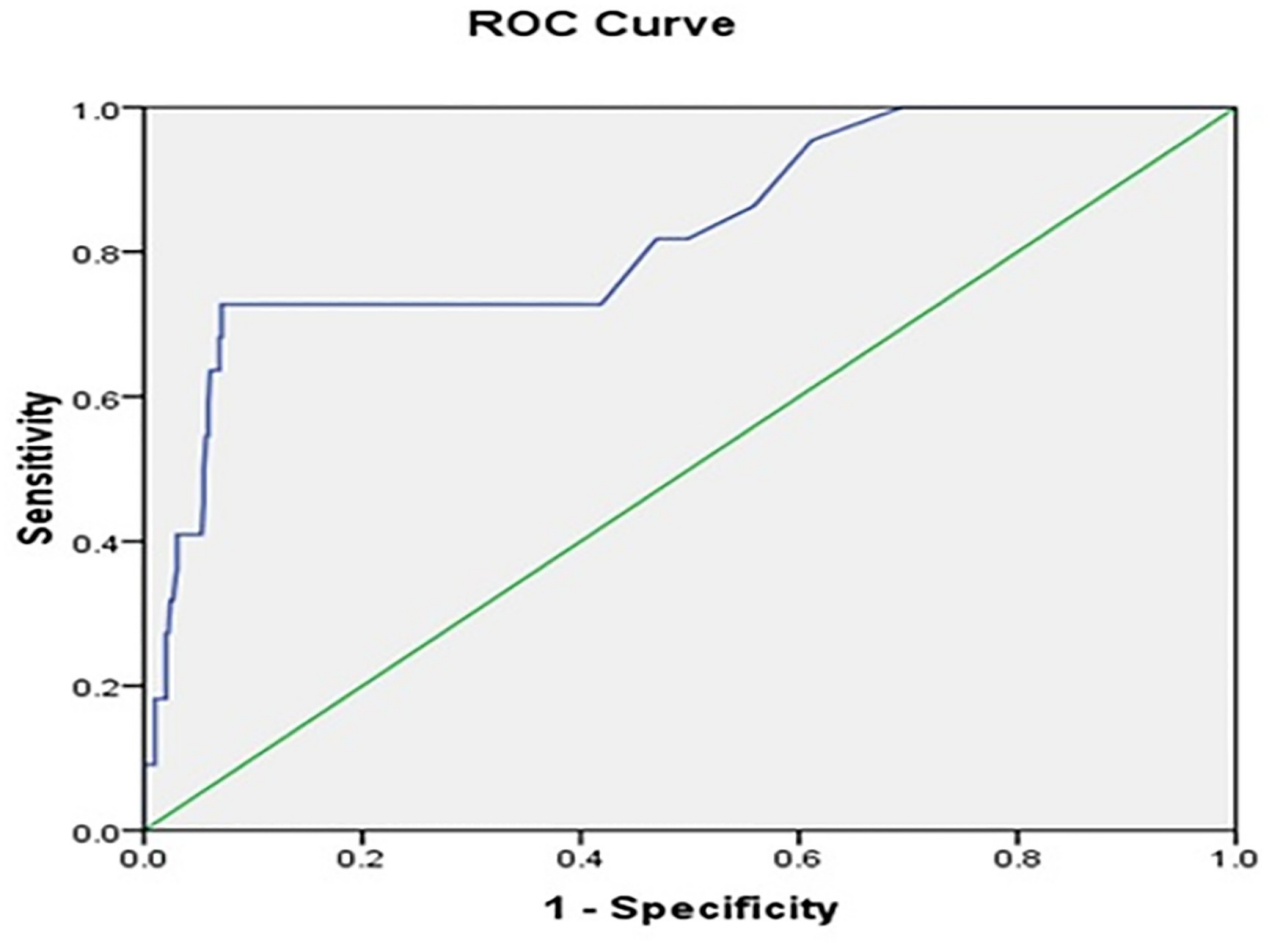
Atypical lymphocyte % more than 0.55 use as a predictive cutoff value to suspect severe dengue cases during the disease progression. (AUC = 87.2% [95%CI = 73%-93.4%]).

Fig 1 show the Receiver Operating Characteristics (ROC) curve analysis of AL% more than 0.55% was predictive cutoff values for severe dengue fever during the disease progression. It has 95.5% sensitivity and 71.5% specificity.

**Fig 2:**
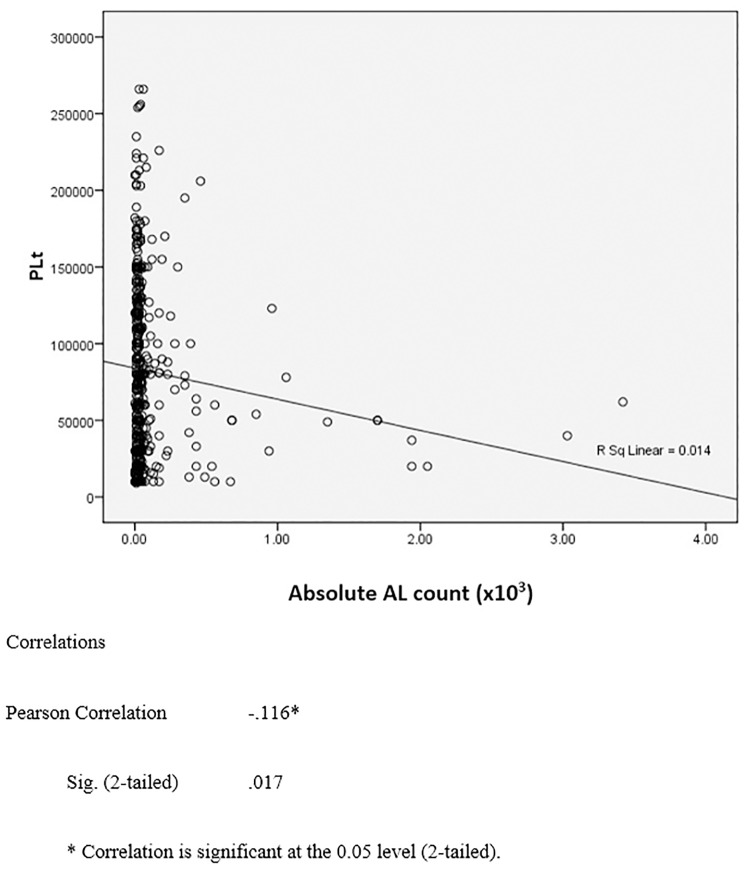
The correlation between platelet count and absolut atypical lymphocyte count (×10^3^).

**Fig 3:**
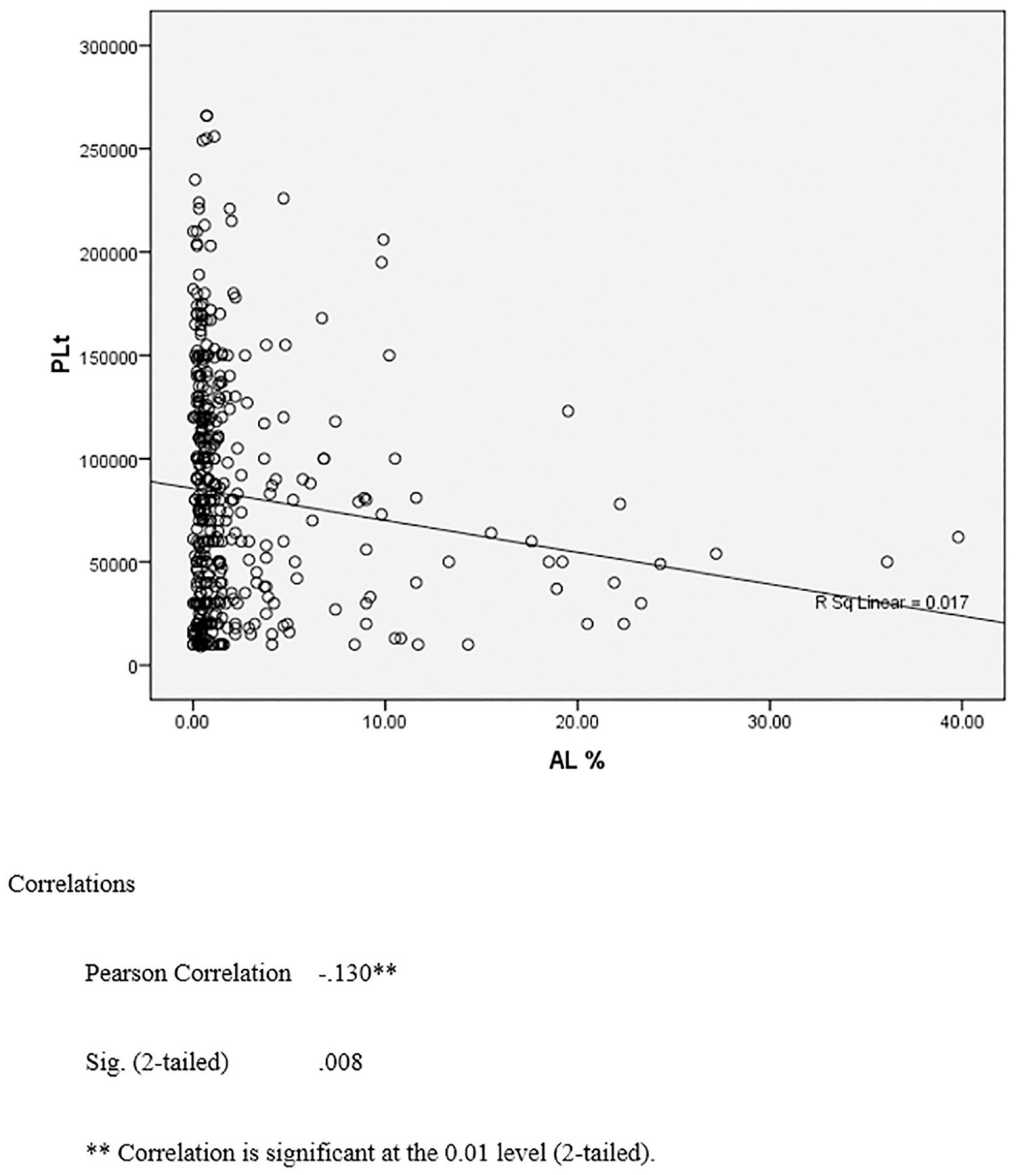
The correlation between platelet count and atypical lymphocyte %.

Based on the Pearson correlation coefficient there was a negative correlation between platelet count and AL%, as well as ALC (×10^3^). (Pearson Correlation = -0.116 [p = 0.017] and Pearson Correlation = -0.130 [p = 0.008]). But R square in the diagrams show only 0.014 and 0.017. (Figs 2 and 3).

## Discussion

We showed that patients with clinical manifestations of severe dengue have a significantly higher AL % compared to dengue without warning signs. Similarly, the AL % among those with respiratory compromise, hypotension and bleeding symptoms is higher as compared to those without. These results suggest that a higher AL % level is associated with increased complications of dengue infection, leading to severe dengue. From Tables 3 and 4, it is evident that patients with severe dengue infection, have significantly more AL % in their peripheral blood, compared to DI without warning signs. In addition, there is a statistically significant 5-fold difference in AL % between severe and non-severe DI. From subgroup analysis between DI with and without warning signs in Table 3, there is a positive association of AL % with severity of DI. The predictive cut-off AL % value for predicting severe DI is > 0.55, as shown in Fig 1, with the sensitivity of 95.5% and specificity of 71.5%. For our study, this is a point estimate which limits its predictive value, therefore, it is likely that consecutive measurements may improve on this and future studies would be required. This is clinically relevant as this may be used as a simple and dynamic predictive measurement for DI severity.

In a study done on the hematological parameters of 138 Indonesian patients, flow cytometry was used to differentiate between DI, leptospirosis and enteric fever. Patients with DI were found to have a higher percentage of AL, when compared to patients with enteric fever and leptospirosis infections [15]. In another study which analyzed the immunophenotypes of lymphocytes in 100 patients with DI of different severity, such as DHF, dengue fever (DF) and dengue like syndrome (DLS), it was concluded that the mean total atypical lymphocytes in DHF and DF were higher than those of DLS[13]. In a recent 2017 study done by Anagha et al [16], they showed that atypical lymphocytes count does predict the severity of thrombocytopenia for DI and this contributes to the severity of dengue. However, this study and other previous similar studies did not investigate the association of AL % with DI clinical parameters such as respiratory failure, shock and bleeding as well as analyze the predictive cut off AL % value for severe DI, which our study did address.

As mentioned earlier, the most critical period of DI is 3–7 days after the onset of the first non-specific symptoms as this is the period where patients develop severe DI complications [17]. Due to the lack of reliable predictive tests, patients with severe DI are at risk of delays in appropriate management, since the majority of DI patients are treated conservatively in outpatient setting. However, if more objective measurements such as AL % can be used to identify these patients, they can be placed on closer monitoring and encouraged for aggressive hydration if early warning signs of severe dengue are present[18].

The increase in AL % is associated with the decrease in platelet count as shown in Table 5 and Fig 3. The analysis of platelet count and AL% has shown that there is an inverse relationship between the two variables, with the correlation coefficient of -0.116. From Table 5, it also demonstrated that with the circulating AL % being more than 20% in the peripheral blood, the mean platelet count could fall below 50 x 10^9^. This may be the explanation for the increase in bleeding complications among dengue patients with higher AL% as demonstrated in Table 2. Thrombocytopenia related to DI is thought to be at least partly immune mediated [19]. It is reasonable to speculate whether the high AL reflects greater immune dysregulation resulting in more severe thrombocytopenia. The same maybe said of the more severe complications of DI. These are important areas to be addressed by future research studies as immunosuppressive therapy such as steroids may warrant evaluation in clinical trials for such patients.

Our study has several limitations. The study population was taken from a single centre in Sri Lanka, therefore the results should be extrapolated with caution to different geographic areas as patients may have a different genetic background, and endemic dengue virus serotypes may differ. Both factors are not controlled for within this study. The population sample data was obtained at different phase of infection and the study has a relatively small number of patients with severe DI. Therefore, these results can be analyzed as a point estimate of the association of AL and severity of DI. This can be improved for future studies by serial analysis of a patient’s FBC throughout the period of DI, from presentation to recovery, as well as include FBC analysis from healthy participants as the control of the study. The Sysmex XS500i automated FBC analyzer may not be readily available in all laboratories in all developing countries, hence, using AL to predict dengue severity may be limited to some institutions. However, we hope that through this study, it demonstrates that routinely performed blood investigations such as full blood count can be used to assess the severity of dengue infection, thus, appealing to a wider audience to participate in using simple and cost-effective measurements for further analysis.

## Conclusions

Clinical manifestations of severe dengue have a significantly higher AL % compared to dengue without warning signs. AL % at presentation may be predictive of severe DI and future larger prospective longitudinal studies should be done to determine if AL % on admission is predictive of the complications of DI later in the course of dengue infection.
